# Supplementary material for: Could Inflammatory Indices and Metabolic Syndrome Predict the Risk of Cancer Development? Analysis from the Bagnacavallo Population Study
Source: J Clin Med. 2020 Apr 20;9(4):1177. doi: 10.3390/jcm9041177 (PMC7231063; doi:10.3390/jcm9041177)
Supplement: Supplementary file 1 [file jcm-09-01177-s001.pdf]

Table S1. List of ICD10 codes and description of tumor site.

ICD10 code	Tumour site
C00-14,30-32	Malignant neoplasm of head and neck
C15	Malignant neoplasm of esophagus
C16	Malignant neoplasm of stomach
C17	Malignant neoplasm of small intestine
C18-21	Malignant neoplasm of colon-rectum
C22	Malignant neoplasm of liver
C25	Malignant neoplasm of pancreas
C33-34	Malignant neoplasm of trachea and lung
C40-41	Malignant neoplasm of bone
C43	Malignant melanoma of skin
C45	Mesothelioma
C46	Kaposi's Sarcoma
C48.0-48.2	Malignant neoplasm of retroperitoneum and peritoneum
C50	Malignant neoplasm of breast
C53	Malignant neoplasm of cervix uteri
C54	Malignant neoplasm of corpus uteri
C55	Malignant neoplasm of cervix nas
C56	Malignant neoplasm of ovary
C58	Malignant neoplasm of placenta
C60	Malignant neoplasm of penis
C61	Malignant neoplasm of prostate
C62	Malignant neoplasm of testis
C64-66,68	Malignant neoplasm of kidney
C67	Malignant neoplasm of bladder
C69-72	Malignant neoplasms of eye, brain and central nervous system
C73	Malignant neoplasm of thyroid
C81	Hodgkin's disease
C82-85,96	non-Hodgkin's lymphoma
C88-90	Multiple Mieloma
C91-95	Leukemia
C00-43,C45-96	All malignant tumors excluding non-melanoma skin
